# The increase in varus tilt of the joint line convergence angle under weight-bearing is correlated with medial meniscus extrusion in patients with knee osteoarthrosis

**DOI:** 10.1371/journal.pone.0331678

**Published:** 2025-09-12

**Authors:** Yosuke Ishii, Akinori Nekomoto, Goki Kamei, Atsuo Nakamae, Takato Hashizume, Kexin Zhu, Miharu Sugimoto, Kohei Matsumura, Makoto Takahashi, Nobuo Adachi

**Affiliations:** 1 Department of Bio-Environmental Adaptation Sciences, Graduate School of Biomedical and Health Sciences, Hiroshima University, Hiroshima, Japan; 2 Department of Orthopaedic Surgery, Graduate School of Biomedical and Health Sciences, Hiroshima University, Hiroshima, Japan; 3 Department of Biomechanics, Graduate School of Biomedical and Health Sciences, Hiroshima University, Hiroshima, Japan; 4 Department of Sports Medical Center, Hiroshima University Hospital, Hiroshima, Japan; Juntendo University, JAPAN

## Abstract

**Background:**

Increased medial meniscus extrusion (MME) in weight-bearing conditions is a critical factor associated with knee osteoarthritis (OA) progression and is not an alternative indicator in nonweight-bearing. This feature is related to mechanical stress. However, its correlation with varus knee alignment, reflecting the loading stress on the medial compartment, is still unknown. This study aimed to determine whether increased MME is associated with varus limb alignment.

**Method:**

Seventy patients with knee OA were recruited for this cross-sectional study. The MME was assessed using ultrasonography. Knee alignments were determined by whole-leg radiography in the standing position, and factors related to the loading stress on the medial compartment of the knee joint were detected, such as the hip-knee-ankle angle, percentage of the mechanical axis, medial proximal tibial angle, and joint line convergence angle (JLCA). MME and JLCA were evaluated under two conditions: nonweight-bearing and weight-bearing, and the increased values were determined as the difference between the conditions (Δ values).

**Results:**

MME and JLCA were significantly higher in the weight-bearing condition than in the nonweight-bearing. The described alignment for MME under nonweight-bearing in the liner model was HKAA, where the ΔMME was ΔJLCA.

**Conclusion:**

The factors of increased MME in weight-bearing conditions differed from those of MME in nonweight-bearing conditions, as shown by the amount of change in JLCA under loading stress.

## Introduction

Medial meniscal extrusion (MME) reflects the destruction of the shock-absorbing function and causes accelerated knee osteoarthritis progression (OA) [1,2]. The MME particularly increases in weight-bearing conditions [3–5]. Increased MME due to weight-bearing (ΔMME) shows a medial meniscus instability [6]. The ΔMME gradually deteriorates with repetitive mechanical stress [7,8], resulting in increased MME [9,10]. Thus, minimizing ΔMME is an ideal prerequisite to prevent knee OA progression and requires a proper approach based on the mechanism of MME.

Limb alignment is a representative indicator of mechanical stress in knee OA [11–13]. Previous studies have shown that varus alignment, including the hip-knee-ankle angle (HKAA), percentage of the mechanical axis (%MA), and medial proximal tibial angle (MPTA) correlates with the extent of MME obtained in the supine position, which is a nonweight-bearing condition [11,13]. However, the extent of MME under nonweight-bearing conditions raises concerns that various situations of daily life, such as walking or standing, are underestimated [14]. Moreover, the nonweight-bearing MME can be easily distinguished from the severity of knee OA [10], whereas the MME itself does not feature progressive knee OA [15]. These reports emphasized that ΔMME is not an alternative indicator to the nonweight-bearing MME, whereas its correlation with knee alignment remains unknown. The ΔMME is correlated with meniscal instability and cartilage damage [6,16]. Therefore, understanding the different correlations in non-weight-bearing MME and ΔMME with varus alignment may contribute to elucidating the mechanism of MME progression and promote to clinical research aimed at clarifying specific factors for the deterioration of MME.

MME occurs due to loading stress, including varus and compression forces on the medial compartment of the tibiofemoral joint [7,8]. In particular, the joint line convergence angle (JLCA) is the varus alignment and shows the gap angle between the tibial and femoral joint lines. This angle reflects the condition of intraarticular arthritis [17] and indicates a greater compression stress on the medial compartment through the combination of MME [18]. Moreover, the JLCA expands under weight-bearing conditions [19,20], similar to a specific MME feature. Therefore, the change in JLCA under loading stress may be strongly correlated with ΔMME which is reflected in the dynamics of MME.

This study aimed to investigate the correlation between knee alignment and ΔMME, and comparison of that with nonweight-bearing MME. The hypothesis was that ΔMME correlates with knee alignment, especially with the difference in JLCA gap with and without weight-bearing, which shows a different correlation with nonweight-bearing MME.

## Materials and methods

### Participants

**Fig 1:**
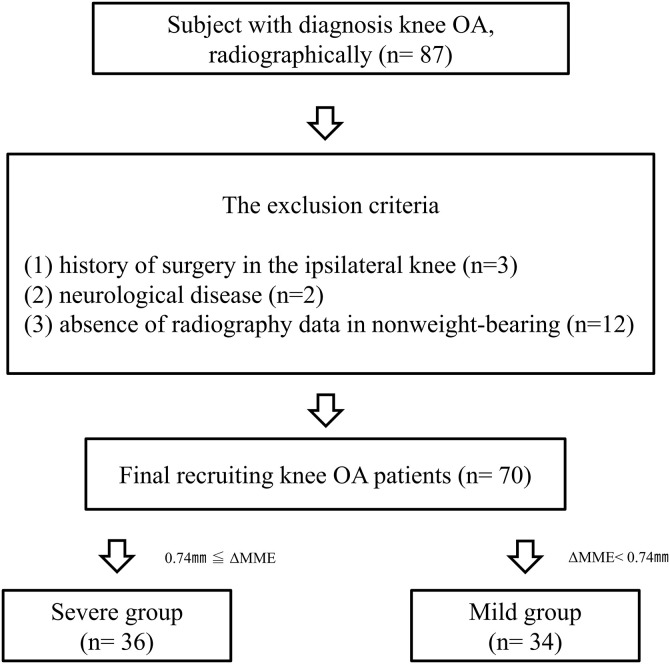
Eligibility protocol for this study.

On October 2022 to December 2024, seventy patients (33 women, mean age 59.3 ± 8.8 years) diagnosed with unilateral or bilateral knee OA participated in this cross-sectional study. Patients with knee OA were recruited preoperatively and underwent around-knee osteotomy or meniscus repair. Sixteen healthy volunteers (4 women, mean age 62.5 ± 7.8 years) were also recruited. The inclusion criteria were the ability to walk smoothly and the presence of arthroscopic findings. The exclusion criteria included (1) a history of surgery in the ipsilateral knee, (2) neurological disease, (3) absence of radiography data in nonweight-bearing **(**Fig 1). The demographic data are shown in Table 1.

**Table 1:** Demographic data and meniscus parameters of the participants.

	*Knee OA*	*mild*	*severe*	*Control*	*p-value*
Knees	70	34	36	16	
Sex (M:F)	33: 37	19: 15	14: 22	12: 4	
KL grade (I/II/III/IV)	11/30/22/7	8/19/6/1	3/11/16/6		
Age (years)	59.3 ± 8.8	57.1 ± 10.4	61.4 ± 6.2	62.3 ± 7.8	0.149
BMI (kg/m²)	26.0 ± 3.6	25.6 ± 3.9*	26.3 ± 3.3*	23.1 ± 2.1	0.001
Supine MME (mm)	4.1 ± 1.8	3.7 ± 1.7*	4.4 ± 1.8*	1.8 ± 0.7	0.001
Standing MME (mm)	4.9 ± 2.0	4.1 ± 1.7*	5.6 ± 1.9*†	2.1 ± 0.8	0.001
ΔMME (mm)	0.8 ± 0.5	0.4 ± 0.2	1.2 ± 0.4*†	0.3 ± 0.3	0.001

BMI, body mass index; K/L grade: Kellgren–Lawrence grade; ΔMME, the difference in MME between nonweight-bearing and weight-bearing. Values are expressed as mean ± standard deviation; p-values indicate significant differences between subgroups and controls. *or † shows significantly higher values than those of the control or mild group (p < 0.05).

This study was approved by the ethics department of our institution in accordance with the Declaration of Helsinki (E449-5). Informed consent was obtained from all participants in writing.

### Evaluation of limb alignment

**Fig 2:**
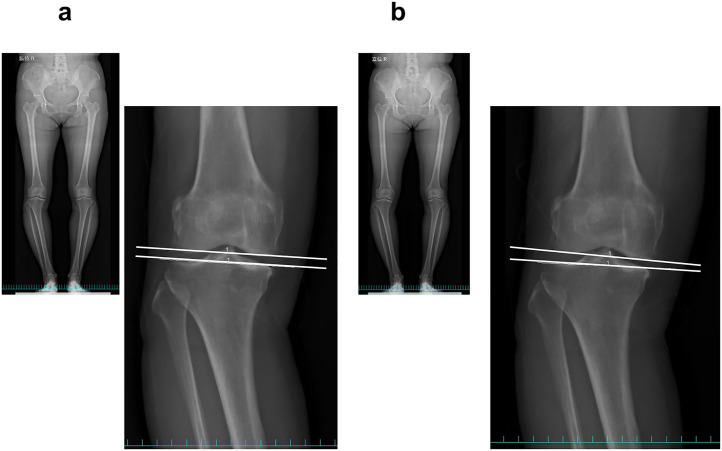
The representative alignment of the joint line convergence angle formed by two white lines, whole-leg radiographs in nonweight-bearing (a) and weight-bearing (b).

Radiographs of participants with knee OA were taken preoperatively under whole-leg conditions with or without weight-bearing. Moreover, the Rosenberg view was assessed knee OA severity using the Kellgren–Lawrence (KL) grading system. Various parameters, including the HKAA, %MA, MPTA, and JLCA, were examined under weight-bearing to evaluate varus knee alignments associated with MME, as indicated in a prior study [11]. Negative HKAA and positive %MA, MPTA, and JLCA values indicated varus alignment. The JLCA was the angle formed by the two tangential lines connecting the medial and lateral femoral condyles with the tibial plateau, and additionally calculated using a nonweight-bearing condition, and the ∆JLCA was determined as the difference between nonweight-bearing and weight-bearing conditions (Fig 2).

A single orthopedic surgeon (N.A.) performed the radiological assessments in a blinded manner to minimize potential bias. In a pilot study [21], the reliability of radiological assessments was confirmed through inter-rater reliability analysis, specifically interclass correlation coefficients (2,1) performed by two examiners. The inter-rater reliability values were as follows: HKAA, 0.986 (95% CI, 0.960–0.995); %MA, 0.914 (95% CI, 0.764–0.970); MPTA, 0.743 (95% CI, 0.391–0.906); and JLCA, 0.743 (95% CI, 0.391–0.906).

### Evaluation of meniscus parameters and categorization into subgroups

**Fig 3:**
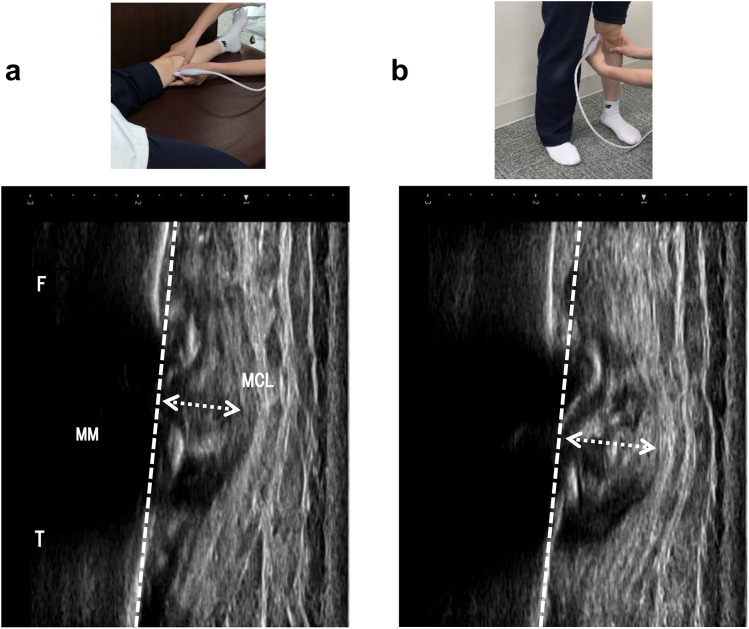
The positions of ultrasound assessment and corresponding images. The ultrasound images of medial meniscus extrusion in nonweight-bearing (a) and weight-bearing **(b)**. MM, medial meniscus; F, femur, T, tibia, MCL, medial collateral ligament. The dashed lines and white double arrows show the line of the medial tibial plateau cortex and the distance of meniscus extrusion.

The MME was assessed preoperatively using an ultrasound device with a linear array transducer (SNiBLE; KONICA MINOLTA, Japan). The measurement protocol adhered to established procedures well-known for their high reliability [22]. The ultrasound transducer was placed longitudinally in the medial joint space to improve image quality at the boundary between the medial meniscus and medial collateral ligament **(**Fig 3) in a supine position and a standing position sustaining weight-bearing stress. The MME allowed precise calculation of the distance from the extruded meniscus, defined as the distance between the cortex of the medial tibial plateau and the outermost edge of the medial meniscus (Figs 3a **and** 3b). This measurement was repeated three times, and Kinovea software (v0.8.15; Kinovea open-source project, https://www.kinovea.org) was used to calculate the MME. The statistically representative value used in the analysis was the average of the three measurements. The ∆MME was determined based on the difference between supine and standing MMEs. The participants were divided into severe or mild groups, using the median ∆MME value as the threshold (Fig 1).

To evaluate the reliability of ΔMME, the standard error of measurement was conducted on six knees using a test-retest measurement over one week. In this pilot study, the standard error of ΔMME was 0.2 mm for intra-rater measurements. Additionally, inter-rater reliability was assessed, yielding a standard error of 0.3 mm.

### Meniscal quality and scoring of the cartilage

The arthroscopic assessment was performed simultaneously by two orthopedic surgeons (G.K. and A.N.) one day after the ultrasonography evaluation of the MME. The medial meniscus condition was recorded as the type and location of injury. The cartilage condition was also scored according to the International Cartilage Repair Society classification.

### Statistical analysis

The entire data set was used to assess the normality of variables. Differences between knee OA subgroups and controls, meniscal parameters, and demographic data were compared using a one-way analysis of variance or the Kruskal-Wallis test. In addition, the cartilage scores within the knee OA subgroups were compared using the Mann-Whitney U test. The correlation between the ΔMME or supine MME and varus alignment was determined using Pearson’s correlation analysis or Spearman’s correlation coefficients. To establish our hypothesis with controlling noise factor using the current sample size, a stepwise multiple regression analysis was conducted with knee alignment as the independent variable to identify the most documented factors for varus alignment correlated with ΔMME and supine MME. All statistical analyses were performed using Statistical Package for the Social Sciences (version 23, IBM, US), and the statistical significance was set at 0.05.

To ensure the validity of the multiple regression analysis with ΔMME, a post-hoc power analysis was conducted using G-power. The power was 94.5% for the sample size.

## Results

### Demographic data and meniscus quality of the participants

This study involved seventy patients with knee OA (Fig 1). The patients with knee OA mainly had mild to moderate severity (KL I:11, II:30, III:22, and IV:7). The demographic data and age did not differ significantly between the groups. The BMIs of the mild and severe groups were higher than those of the control group (Table 1). For meniscus quality, patients with knee OA showed the most frequent degenerative tears (40%). Moreover, the medial meniscus posterior root tear (MMPRT) was present in 21% of all knee OA (Table 2).

**Table 2:** Cartilage and meniscus parameters.

	*Knee OA*	*mild*	*severe*	*p-value*
ICRS score	2.9 ± 1.4	2.5 ± 1.5	3.3 ± 1.1	0.032
Meniscus tear				
Intact (%)	1 (1)	0 (0)	1 (3)	
Radial (%)	9 (13)	1 (3)	8 (22)	
Longitudinal (%)	2 (3)	2 (6)	0 (0)	
Horizontal (%)	6 (9)	3 (9)	3 (8)	
Degenerative (%)	28 (40)	15(44)	13(36)	
Complex (%)	9 (13)	5 (15)	4 (11)	
MMPRT (%)	15 (21)	8 (24)	7 (19)	

ICRS, International Cartilage Repair Society; MMPRT, medial meniscus posterior root tear.

### Meniscal extrusion and cartilage lesion

In all groups, the standing MME was significantly higher than the supine MME. The ∆MME was 0.8 ± 0.5 mm in the knee OA patients. The ∆MME did not correlate significantly with supine MME (r = 0.214, p = 0.074). The ∆MME in the severe group was higher than that in the mild and control groups. The significant difference was not observed between the mild and control groups (∆MME in the severe group: 1.2 ± 0.4 mm, mild: 0.4 ± 0.2 mm, control: 0.3 ± 0.3 mm; severe vs mild, p = 0.001, **d* *= 2.43; severe vs control, p = 0.001, **d* *= 2.56; mild vs control, p = 0.458, **d* *= 0.44) (Table 1). The cartilage score was significantly higher in the severe group than in the mild group (severe: 3.3 ± 1.1; mild: 2.5 ± 1.5; p = 0.032, **r* *= 0.26) (Table 2).

### Correlation between limb alignments, MME, and ∆MME

Limb alignment under weight-bearing data were shown in **Table 3**. Weight-bearing JLCA was significantly higher than nonweight-bearing JLCA (nonweight-bearing: 1.6 ± 1.1 degrees, weight-bearing: 2.4 ± 1.3 degrees), and *∆JLCA was* 0.8 ± 0.6 degrees.

**Table 3:** Limb alignments under weight-bearing of patients with knee OA.

	Knee OA
HKAA	−5.5 ± 3.8
%MA	25.7 ± 14.7
MPTA	84.0 ± 2.0
JLCA	2.4 ± 1.3
Non-weight-bearing JLCA	1.6 ± 1.1
ΔJLCA	0.8 ± 0.6

HKAA, hip-knee-ankle angle; %MA, percentage of the mechanical axis; MPTA, medial proximal tibial angle; JLCA, joint line convergence angle; ΔJLCA, the difference in JLCA between nonweight-bearing and weight-bearing Values represent means ± standard deviation.

Correlation analysis with supine MME showed that HKAA, %MA and MTPA were negatively correlated with MME (HKAA: r = −0.567, p = 0.001, %MA: r = −0.567, p = 0.001, MTPA: r = −0.23, p = 0.049). In contrast, JLCA with and without weight-bearing showed positive significant correlations (nonweight-bearing JLCA: r = 0.43, p = 0.001, weight-bearing JLCA: r = 0.53, p = 0.001), but those correlations were not shown in ∆JLCA (Table 4).

**Table 4:** Correlations between ΔMME, MME, and knee alignments.

	MME	*p-*value	ΔMME	*p-*value
HKAA	−0.567	0.001	−0.349	0.03
%MA	−0.567	0.001	−0.362	0.002
MPTA	−0.23	0.049	−0.26	0.028
JLCA	0.53	0.001	0.49	0.001
Non-weight bearing JLCA	0.43	0.001	0.32	0.001
ΔJLCA	0.116	0.334	0.56	0.001

HKAA, hip-knee-ankle angle; %MA, percentage of mechanical axis; MPTA, medial proximal tibial angle; JLCA, joint line convergence angle; ΔJLCA, the difference in JLCA between nonweight-bearing and weight-bearing. Values represent correlation coefficients; p-values show significant correlations between knee alignments and MME or ΔMME.

For ∆MME, the correlation analysis showed that both HKAA, %MA and MTPA were negatively correlated with ∆MME (HKAA: r = −0.349, p = 0.03, %MA: r = −0.362, p = 0.002, MTPA: r = −0.26, p = 0.028). In contrast, JLCAs and ∆JLCA showed positive significant correlations (nonweight-bearing JLCA: r = 0.32, p = 0.001, weight-bearing JLCA: r = 0.49, p = 0.001, ∆JLCA: r = 0.56, p = 0.001) (Table 4).

### Multiple regression analysis of MME and ∆MME

The results of the multiple regression analysis are shown in Tables 5 and 6. The independent variables were identified as MME or ∆MME. The MME was stronger when comparing Model 2 (adjusted R^2 ^= 0.395, p = 0.002) with Model 1 (adjusted R^2 ^= 0.312, p = 0.001). Model 1 was showed HKAA (β: −0.26, p = 0.001). Model 2 included HKAA and weight-bearing JLCA (β, HKAA: −0.19, p = 0.001; weight-bearing JLCA: 0.468, p = 0.002) (**Table 5**).

**Table 5:** Multiple regression analysis for MME as the knee alignment.

	Standard β	p-value	R	R^2^	Adjusted R^2^
Model 1		<0.01	0.568	0.322	0.312
HKAA	−0.264	<0.01			
Model 2		<0.01	0.642	0.412	0.395
HKAA	−0.19	<0.01			
JLCA	0.468	<0.01			

HKAA, hip-knee-ankle angle; JLCA, joint line convergence angle.

**Table 6:** Multiple regression analysis for ΔMME as the knee alignment.

	Standard β	p-value	R	R^2^	Adjusted R^2^
Model 1		<0.01	0.568	0.323	0.313
ΔJLCA	0.477	<0.01			
Model 2		<0.01	0.637	0.406	0.388
ΔJLCA	0.367	<0.01			
JLCA	0.12	<0.01			

JLCA, joint line convergence angle; ΔJLCA, the difference in JLCA between nonweight-bearing and weight-bearing.

The ∆MME was stronger when comparing Model 2 (adjusted R^2 ^= 0.388, p = 0.003) with Model 1(adjusted R^2 ^= 0.313 p = 0.001). Model 1 showed ∆JLCA (β: 0.477, p = 0.001). Model 2 included JLCA and ∆JLCA (β, JLCA: 0.12, p = 0.001; ∆JLCA: 0.367, p = 0.001) (**Table 6**).

## Discussion

This is the first investigation of the correlation between increased MME and varus knee alignment, and comparison of that with nonweight-bearing MME by weight-bearing. Our results suggest that the difference in JLCA between patients with weight-bearing and nonweight-bearing better predicts the increase in MME, whereas the nonweight-bearing MME describes HKAA. These results were in line with our hypothesis, which has different correlations in each nonweight-bearing MME and an increase in MME.

Our data showed that the linear model of ∆MME was mostly of ∆JLCA, while the MME in the nonweight-bearing was HKAA or JLCA. Moreover, the ∆MME was not significantly correlated with nonweight-bearing MME. Thus, the ∆MME has a different pathology from the nonweight-bearing MME in terms of the underlying mechanical stress. The JLCA is interpreted as the severity of knee OA [17]. Thus, the nonweight-bearing MME and varus knee alignment, including JLCA and HKAA, are common in that they gradually worsen according to knee OA severity [10,17], and their correlations with limb alignment were partially consistent with a previous study [11]. On the other hand, the ∆JLCA was the most documented factor describing the ∆MME. On the prediction of ∆MME, the value of coefficient of determination on ∆JLCA model was 0.313. Even when JLCA was included as an additional variable, the value of the coefficient of determination only slightly increased to 0.388, which did not almostly change. Moreover, there was a sufficient statistical power of 94.5% on ∆JLCA model. These findings indicate that the ∆JLCA is a favorite factor to describe of ∆MME. The JLCA strongly depends on the width of the medial joint space [23]. A previous study found a simultaneous increase in MME and narrowing of the width in the medial joint space in patients with knee OA under weight-bearing, with a greater response than in healthy subjects [24]. Therefore, these previous studies could explain the rational correlation in changes under load stress between the medial meniscus and alignment, including the width in the medial joint space.

Increased MME correlates with meniscal instability and cartilage damage [6,16]. Our data showed that in the severe group with greater ΔMME, the International Cartilage Repair Society score was higher than that in the mild group. The standard error of ΔMME was 0.2 to 0.3 mm, indicating it was smaller than the threshold value between the severe and mild groups (median: 0.74 mm), which had potential that the accuracy of ultrasonography evaluation did not directory distort our result. Murakami et al. demonstrated the longitudinal study in early and mild stages of knee OA and showed that progressive knee OA with worsening MME after follow-up had the feature of high ΔMME at baseline but not that of MME itself [15]. This could explain why the ΔMME causes greater MME, resulting in knee OA progression. Therefore, acquiring the minimum ΔMME is valuable for preventing knee OA progression, indicating the need to explore the underlying mechanism.

MMPRT is known to directly cause MME owing to the destruction of the hoop structure of the meniscus [25,26]. Interestingly, although our data included some patients with MMPRT, almost all patients had a greater MME (>3 mm). Moreover, in the severe group, which had a high ΔMME, the meniscal quality did not differ from that of the mild group. This provides insight into another factor that worsens the MME without an abnormal hoop structure. Varus alignment indicates mechanical stress in the medial compartment [11,13,27]. However, it remains unknown which mechanical stress leads to increased MME. Our findings showed that ΔJLCA correlated with ΔMME, an indicator for knee OA progression, and that the mechanism of MME differs in nonweight-bearing. This is the first step to understanding the detailed mechanics of MME and would support further longitudinal study with a hypothesis on clear mechanical factors. Therefore, these previous studies and our findings provide a mechanism for the increase in MME under mechanical stress and lead to the development of an approach to prevent knee OA progression.

This study has several limitations. First, this study had a small sample size and, therefore, inadequately analyzed meniscal features, such as the type or location of injury, especially using the limited statistical model of the stepwise method, whereas this technique expects to control the noise factor with sufficient statistical power, which this process could not directory distort our findings. Second, this study was cross-sectional; thus, it was challenging to identify the leading cause or effect of meniscal extrusion and knee alignment with weight-bearing. However, high tibial osteotomy reduces MME due to postoperative changes of loading stress [21]. This may support the hypothesis that mechanical stress due to knee alignment causes MME during weight-bearing. Further studies are needed to provide a detailed analysis of cohort studies with sufficient sample size.

## Conclusion

The described factor of the increase in the MME differed from that in the MME and was shown by the amount of JLCA change under loading stress.

## Abbreviations

MME: Medial meniscus extrusion
OA: Osteoarthritis
HKAA: Hip knee ankle angle
%MA: Percentage of the mechanical axis
MTPA: Medial proximal tibial angle
JLCA: Joint line convergence angle
MMPRT: Medial meniscus posterior root tear
ΔMME: The difference in medial meniscus extrusion between nonweight-bearing and weight-bearing
ΔJLCA: the difference in joint line convergence angle between nonweight-bearing and weight-bearing.
