# Correction: The increase in varus tilt of the joint line convergence angle under weight-bearing is correlated with medial meniscus extrusion in patients with knee osteoarthrosis

**DOI:** 10.1371/journal.pone.0350703

**Published:** 2026-06-04

**Authors:** The PLOS One Editors

## Notice of Republication

This article [1] was republished on April 16, 2026, to address an issue identified post-publication. An updated version of S1 Data is provided with this notice. Please download this article again to view the correct version.

The article’s Data Availability statement is updated to: All relevant data are within the paper and its Supporting Information files.

## Supporting information

S1 Data Raw data underlying Figs 1–3 and Tables 1–6. (XLSX)
